# Sulfur microbial diet and ischemic stroke risk

**DOI:** 10.3389/fnut.2026.1885612

**Published:** 2026-10-22

**Authors:** Kai Wang, Zhiyuan Guo, Liyuan Han, Boding Wang

**Affiliations:** 1 Department of Neurosurgery, Ningbo No. 2 Hospital, Wenzhou Medical University, Ningbo, Zhejiang, China; 2 School of Public Health, Wannan Medical College, Wuhu, China; 3 Department of Clinical Epidemiology, Ningbo No. 2 Hospital, Wenzhou Medical University, Ningbo, Zhejiang, China; 4 Center for Cardiovascular and Cerebrovascular Epidemiology and Translational Medicine, Guoke Ningbo Life Science and Health Industry Research Institute, University of Chinese Academy of Sciences, Ningbo, Zhejiang, China

**Keywords:** dose-response, gut microbiota, ischemic stroke, prospective cohort study, sulfur microbial diet

## Abstract

**Background:**

This study aimed to evaluate the association of dietary pattern linked to sulfur-metabolizing gut microbiota (SMD) with incident ischemic stroke, its dose–response relationship, and population heterogeneity.

**Methods:**

This study included 208,415 participants. The SMD score was constructed based on repeated 24-h dietary recall questionnaires (Oxford WebQ) and categorized into quartiles. Cox proportional hazards models were used to estimate hazard ratios (HRs) and 95% confidence intervals (CIs). Restricted cubic spline analyses were performed to assess the dose–response relationship. Subgroup analyses, sensitivity analyses, and food substitution analyses were further conducted.

**Results:**

During a mean follow-up of 11.45 years, a total of 3,273 incident ischemic stroke cases were identified. After full multivariable adjustment, compared with participants in the lowest quartile (Q1), those in the highest quartile (Q4) had a 13% higher hazard of ischemic stroke (HR = 1.13, 95% CI: 1.02, 1.25, *p* = 0.019). Each 1-standard deviation increase in the SMD score was associated with a 4% higher hazard of ischemic stroke (HR = 1.04, 95% CI: 1.01, 1.08, *p* = 0.018), with a significant linear trend (*P* for trend = 0.013). Restricted cubic spline analysis suggested an approximately linear association (*P* for nonlinearity = 0.232). Subgroup analyses showed heterogeneity by ethnicity (*P* for interaction = 0.003), whereas interactions by age and sex were not statistically significant. Sensitivity analyses yielded generally consistent estimates. Substitution analysis indicated an HR below 1 when low-calorie beverages were replaced with an equivalent energy intake of other vegetables (HR = 0.90, 95% CI: 0.81, 1.01, *p* = 0.063).

**Conclusion:**

Higher adherence to the SMD was associated with a higher hazard of ischemic stroke in this large prospective cohort, with an approximately linear dose–response relationship. The association was modest and showed some sensitivity to exposure measurement precision, indicating that replication in independent cohorts is warranted.

## 1 Introduction

Ischemic stroke is one of the leading causes of death and disability worldwide. Owing to its insidious onset, poor prognosis, and high recurrence rate, it has become a major public health challenge (1, 2). Although traditional risk factors such as hypertension, diabetes mellitus, dyslipidemia, and smoking have been well established, a considerable proportion of stroke events cannot be fully explained by these known factors, suggesting the existence of additional modifiable determinants that remain insufficiently characterized (3, 4). In recent years, with the growing understanding of the gut-brain axis, increasing attention has been paid to the role of the gut microbiota and its metabolites in cerebrovascular diseases (5). As the most fundamental and modifiable environmental factor shaping the gut microbiota, diet serves as a critical link connecting dietary patterns, gut microbial ecology, and stroke risk (6, 7).

It is important to note that we did not measure the gut microbiome or sulfur metabolites directly. Instead, we used a dietary index (SMD) previously developed and validated against metagenomic data, which characterizes dietary patterns linked to the functional capacity of sulfur-metabolizing gut bacteria (8). It is characterized by higher intakes of processed meats, alcohol, and low-calorie beverages, and lower intakes of legumes, vegetables, natural fruit products, and certain protective foods. Previous studies have suggested that this dietary pattern may promote the enrichment of sulfate-reducing bacteria in the gut and increase the production of sulfur-containing metabolites such as hydrogen sulfide (9, 10), thereby impairing the intestinal mucosal barrier, inducing systemic chronic low-grade inflammation, aggravating metabolic dysfunction, and damaging vascular endothelial function (11). Existing epidemiological evidence has linked the SMD to multiple adverse health outcomes, including colorectal cancer, obesity, nonalcoholic fatty liver disease, chronic kidney disease, and all-cause mortality (12). However, its association with ischemic stroke has not yet been adequately examined in large prospective cohorts (13). Although current pathophysiological evidence suggests that the SMD may promote stroke development through inflammation, oxidative stress, metabolic disturbance, and atherosclerosis, direct support from large-scale longitudinal population studies remains lacking.

Meanwhile, previous epidemiological studies on diet and stroke have several important limitations. Most have focused on individual nutrients or traditional dietary indices, such as the Mediterranean diet and the Dietary Approaches to Stop Hypertension (DASH) diet, while few have explored gut microbiota-specific dietary patterns. Many studies have employed cross-sectional or retrospective designs, limiting the establishment of temporality. Dietary exposure assessment has often relied on a single dietary questionnaire, which is prone to measurement error. Moreover, subgroup analyses and substitution analyses have rarely been conducted. More importantly, no previous study has simultaneously addressed two key scientific questions: first, whether the SMD is associated with incident ischemic stroke; and second, whether this association exhibits a linear dose–response relationship and population heterogeneity.

Using repeated 24-h dietary recall data to construct a standardized SMD score, we evaluated its association with incident ischemic stroke after adjustment for potential confounders. We further examined the dose–response relationship, population heterogeneity, and exploratory food substitution estimates.

## 2 Methods

### 2.1 Study design and study population

This study is a large-scale, population-based prospective cohort study that enrolled more than 500,000 participants aged 40–69 years across the United Kingdom between 2006 and 2010 (14). Participants were recruited from 22 assessment centers across England, Scotland, and Wales, with baseline questionnaire data, physical measurements, and biological samples collected. For the present study, participants were included if they had at least one valid 24-h dietary recall assessment, information required to calculate the SMD score, no prior cerebrovascular disease at the dietary index date (ICD-10 codes I60–I64, G45, and I69), and available follow-up. The dietary index date was defined as the date of the last valid dietary recall included in the participant-specific mean and served as the follow-up origin. Participants with prior cerebrovascular disease at the dietary index date, unreliable dietary data, missing SMD component data, or incomplete follow-up were excluded. Unreliable dietary data included energy intake below 500 kcal/day or above 4,500 kcal/day, more than 50% missing dietary items, or an invalid recall flag. Participants with at least one valid 24-h dietary recall were included in the primary analysis, yielding 208,415 participants (Supplementary Figure S1). The Cox regression, restricted cubic spline, subgroup, and food substitution analyses were conducted in this primary analytic cohort, and the sensitivity analyses were conducted in prespecified subsets of it. This study was approved by the North West Multi-centre Research Ethics Committee as a Research Tissue Bank (REC reference 16/NW/0274), and all participants provided written informed consent. This study was conducted under application 76,819.

### 2.2 Construction of the sulfur microbial diet score

Dietary data were obtained from repeated 24-h dietary recall questionnaires using the Oxford WebQ, a validated tool for dietary assessment in large-scale population studies (15). Mean intake across valid recalls was used to estimate each participant’s food-group intake. The SMD score followed the published derivation algorithm (8) and comprised eight food groups: processed meat, low-calorie beverages, and liquor/spirits with positive coefficients; and beer/cider, fruit juice, legumes, other vegetables, and sweets/desserts with negative coefficients. For each component, intake was standardized within the analytic population as *z* = (individual intake − population mean) / population standard deviation. The component z scores were multiplied by the published coefficients (0.64, 0.38, 0.31, −0.54, −0.21, −0.64, −0.30, and −0.23, respectively) and summed. Higher scores indicated greater adherence to the SMD pattern. The SMD score was analyzed in quartiles (Q1–Q4) and per 1-standard deviation increment.

### 2.3 Outcome ascertainment

The primary outcome was incident ischemic stroke (ICD-10 I63), ascertained through linked hospital admission records and death registry data. Follow-up began on the date of the last valid dietary recall included in the participant-specific mean and continued until the first ischemic stroke, all-cause death, loss to follow-up, or the end of follow-up, whichever occurred first. Participants who developed hemorrhagic stroke, other stroke subtypes, or died from non-stroke causes were censored at the corresponding time point.

### 2.4 Covariates

Covariates were selected on the basis of prior literature, clinical relevance, and their temporal relation to the dietary exposure and outcome. Demographic and socioeconomic variables included age, sex, ethnicity, Townsend deprivation index, educational attainment, annual household income, and employment status. Lifestyle variables included smoking status, alcohol drinking frequency, physical activity, and total energy intake. For Cox regression Models 1–3, we used composite definitions that additionally incorporated medication indicators: Hypertension was defined as systolic blood pressure ≥ 140 mmHg, diastolic blood pressure ≥ 90 mmHg, or use of blood-pressure-lowering medication. Diabetes was defined as doctor-diagnosed diabetes, insulin use, HbA1c ≥ 48 mmol/mol, or glucose ≥ 7 mmol/L. Medication indicators contributed to these definitions and were not entered again as separate covariates in models containing hypertension or diabetes. Physical activity was expressed as metabolic equivalent of task minutes per week.

### 2.5 Statistical analysis

All statistical analyses were performed using R software and were based on multiply imputed datasets. Baseline characteristics were summarized across SMD quartiles; continuous variables are presented as mean +/− standard deviation and categorical variables as number (percentage). Overall differences across quartiles were assessed using one-way analysis of variance for continuous variables and Pearson’s chi-square test for categorical variables. Cox proportional hazards models were used to estimate HRs and 95% CIs. Model 1 was adjusted for age, sex, and ethnicity. Model 2 was further adjusted for BMI, Townsend deprivation index, education, household income, employment status, smoking status, alcohol drinking frequency, physical activity, diabetes, coronary heart disease, and hypertension. Model 3 was further adjusted for total energy intake. HRs were reported across SMD quartiles and per 1-standard deviation increment, linear trends were tested, and the proportional hazards assumption was assessed.

Missing covariate data were handled using multiple imputation by chained equations implemented with the R package mice (*m* = 5; maxit = 5). Predictive mean matching was used for continuous variables, logistic regression for binary categorical variables, and polytomous logistic regression for unordered multicategorical variables. The imputation model included the SMD score, age, sex, ethnicity, BMI, Townsend deprivation index, education, household income, employment, smoking status, alcohol status, physical activity, coronary heart disease, hypertension-related variables, diabetes-related variables, medication-related variables, total energy intake, the ischemic-stroke event indicator, and follow-up information. The SMD exposure, SMD component exposure variables, ischemic-stroke event status and event date, and follow-up/index-date variables were not imputed. Cox proportional hazards models were fitted separately in each of the five completed datasets, and regression coefficients and corresponding variance estimates were combined using Rubin’s rules.

Restricted cubic spline analyses assessed dose–response shape and potential nonlinearity. Subgroup analyses were conducted according to age, sex, ethnicity, alcohol drinking status, and smoking status. Sensitivity analyses excluded events within the first 2 years, participants with only one dietary recall, and participants with baseline diabetes or coronary heart disease. Isocaloric substitution models evaluated replacement of specific food components. In Cox regression Models 1–3, hypertension and diabetes were defined using the composite definitions shown in Supplementary Table S3 (i.e., including medication indicators). Table 1, by contrast, used baseline descriptive definitions based on measured values and/or diagnoses at enrollment for the purpose of characterizing the cohort at baseline.

**Table 1:** Baseline characteristics of participants according to SMD quartiles.

Variable	Total	Q1	Q2	Q3	Q4	*p*-value
Age (mean ± SD)	56.08 ± 7.95	56.59 ± 7.78	56.48 ± 7.85	56.10 ± 7.98	55.16 ± 8.10	<0.001
Sex, *n* (%)		<0.001				
Female	114,866 (55.11)	29,229 (56.06)	29,365 (56.37)	29,203 (56.04)	27,069 (51.98)	
Male	93,549 (44.89)	22,913 (43.94)	22,724 (43.63)	22,908 (43.96)	25,004 (48.02)	
Ethnicity, *n* (%)		<0.001				
Non-white	8,748 (4.20)	1,726 (3.31)	1,879 (3.61)	2,317 (4.45)	2,826 (5.43)	
White	199,667 (95.80)	50,416 (96.69)	50,210 (96.39)	49,794 (95.55)	49,247 (94.57)	
BMI (mean ± SD)	26.95 ± 4.65	26.50 ± 4.51	26.65 ± 4.49	26.90 ± 4.57	27.75 ± 4.93	<0.001
Townsend (mean ± SD)	−1.58 ± 2.87	−1.58 ± 2.85	−1.68 ± 2.82	−1.61 ± 2.86	−1.44 ± 2.96	<0.001
Education, *n* (%)		<0.001				
A/O levels or equivalent	70,550 (33.85)	16,894 (32.40)	17,430 (33.46)	17,826 (34.21)	18,400 (35.34)	
CSE/NVQ/HND/HNC/OTHER	30,791 (14.77)	7,173 (13.76)	7,253 (13.92)	7,730 (14.83)	8,635 (16.58)	
College/University degree	89,241 (42.82)	24,227 (46.46)	23,327 (44.78)	21,976 (42.17)	19,710 (37.85)	
None of the above	17,833 (8.56)	3,848 (7.38)	4,079 (7.83)	4,578 (8.79)	5,328 (10.23)	
Income, *n* (%)		<0.001				
18,000–30,999	51,522 (24.72)	13,304 (25.52)	12,956 (24.87)	12,792 (24.55)	12,469 (23.95)	
31,000–51,999	58,421 (28.03)	14,576 (27.95)	14,713 (28.25)	14,608 (28.03)	14,523 (27.89)	
52,000–100,000	49,249 (23.63)	11,979 (22.97)	12,493 (23.98)	12,332 (23.66)	12,445 (23.90)	
Greater than 100,000	14,952 (7.17)	3,541 (6.79)	3,778 (7.25)	3,833 (7.36)	3,800 (7.30)	
Less than 18,000	34,272 (16.44)	8,742 (16.77)	8,149 (15.64)	8,546 (16.40)	8,835 (16.97)	
Employment, *n* (%)		<0.001				
Employed	127,036 (60.95)	30,566 (58.62)	30,943 (59.40)	31,815 (61.05)	33,712 (64.74)	
Not employed	13,705 (6.58)	3,371 (6.46)	3,285 (6.31)	3,369 (6.47)	3,681 (7.07)	
Other	1,784 (0.86)	514 (0.99)	463 (0.89)	423 (0.81)	384 (0.74)	
Retired	65,889 (31.61)	17,691 (33.93)	17,398 (33.40)	16,503 (31.67)	14,297 (27.45)	
Smoking, *n* (%)		<0.001				
Current	16,307 (7.82)	3,462 (6.64)	3,625 (6.96)	4,036 (7.74)	5,185 (9.96)	
Former	74,139 (35.57)	19,262 (36.94)	18,352 (35.23)	18,001 (34.54)	18,524 (35.57)	
Never	117,969 (56.60)	29,418 (56.42)	30,112 (57.81)	30,074 (57.71)	28,364 (54.47)	
Alcohol, *n* (%)		<0.001				
Daily or almost daily	47,756 (22.91)	12,176 (23.35)	12,283 (23.58)	11,788 (22.62)	11,509 (22.10)	
Never	13,073 (6.27)	3,248 (6.23)	2,943 (5.65)	3,303 (6.34)	3,579 (6.87)	
Once or twice a week	51,842 (24.87)	12,819 (24.59)	12,816 (24.60)	13,172 (25.28)	13,035 (25.03)	
One to three times a month	22,957 (11.02)	5,440 (10.43)	5,662 (10.87)	5,714 (10.97)	6,142 (11.79)	
Special occasions only	20,606 (9.89)	4,977 (9.55)	4,895 (9.40)	5,198 (9.97)	5,536 (10.63)	
Three or four times a week	52,180 (25.04)	13,481 (25.85)	13,490 (25.90)	12,937 (24.83)	12,271 (23.57)	
Physical activity, *n* (%)		<0.001				
High	47,794 (22.93)	13,542 (25.97)	11,940 (22.92)	11,563 (22.19)	10,749 (20.64)	
Low	76,561 (36.73)	17,301 (33.18)	18,917 (36.32)	19,593 (37.60)	20,750 (39.85)	
Moderate	84,061 (40.33)	21,299 (40.85)	21,232 (40.76)	20,956 (40.21)	20,574 (39.51)	
Diabetes, *n* (%)		<0.001				
No	199,680 (95.81)	50,419 (96.70)	50,125 (96.23)	49,975 (95.90)	49,161 (94.41)	
Yes	8,735 (4.19)	1,723 (3.30)	1,964 (3.77)	2,136 (4.10)	2,912 (5.59)	
CHD, *n* (%)		<0.001				
No	201,433 (96.65)	50,508 (96.87)	50,463 (96.88)	50,391 (96.70)	50,071 (96.16)	
Yes	6,982 (3.35)	1,634 (3.13)	1,626 (3.12)	1,720 (3.30)	2,002 (3.84)	
Hypertension, *n* (%)		<0.001				
No	157,684 (75.66)	39,786 (76.30)	39,819 (76.44)	39,691 (76.17)	38,388 (73.72)	
Yes	50,731 (24.34)	12,356 (23.70)	12,270 (23.56)	12,420 (23.83)	13,685 (26.28)	
Blood pressure medication, *n* (%)		<0.001				
No	187,267 (89.85)	47,084 (90.30)	47,113 (90.45)	47,038 (90.26)	46,032 (88.40)	
Yes	21,148 (10.15)	5,058 (9.70)	4,976 (9.55)	5,073 (9.74)	6,041 (11.60)	
Insulin, *n* (%)		<0.001				
No	207,694 (99.65)	52,004 (99.73)	51,928 (99.69)	51,918 (99.63)	51,845 (99.56)	
Yes	721 (0.35)	138 (0.27)	161 (0.31)	193 (0.37)	228 (0.44)	
Total energy intake (mean ± SD)	8870.15 ± 2721.15	9688.85 ± 2805.01	9011.20 ± 2544.01	8561.85 ± 2574.93	8217.79 ± 2727.13	<0.001

Data are presented as mean ± SD for continuous variables and *n* (%) for categorical variables. Values were summarized across five multiply imputed datasets generated using multiple imputation by chained equations. SMD, sulfur microbial diet score; BMI, body mass index; CHD, coronary heart disease; SD, standard deviation. Overall *p*-values were calculated using one-way analysis of variance for continuous variables and Pearson’s chi-square test for categorical variables. These baseline definitions do not incorporate medication use and differ from the composite definitions used in all Cox regression models (see Methods and Supplementary Table S3), which additionally include blood-pressure-lowering medication and insulin use to capture participants with controlled disease.

## 3 Results

### 3.1 Baseline characteristics of the study population

A total of 208,415 participants were included. Baseline characteristics differed across SMD quartiles. Compared with Q1, participants in Q4 were younger and more often male and of non-White ethnicity; they also had higher BMI, a higher prevalence of hypertension, diabetes mellitus, and coronary heart disease, lower physical activity, a higher prevalence of current smoking, and lower total energy intake. Overall group-comparison *p*-values are reported in Table 1.

### 3.2 Association between the sulfur microbial diet score and ischemic stroke

During a mean follow-up of 11.45 years, 3,273 incident ischemic stroke cases were identified. Cox proportional hazards models showed a significant positive association between the SMD and ischemic stroke risk. In the fully adjusted model (Model 3), compared with Q1, the HR for ischemic stroke in Q4 was 1.13 (95% CI: 1.02, 1.25; *p* = 0.019). When analyzed as a continuous variable, each 1-standard deviation increase in the SMD score was associated with a 4% increase in the risk of ischemic stroke (HR = 1.04, 95% CI: 1.01, 1.08; *p* = 0.018), with a significant linear trend (*P* for trend = 0.013). Across progressively adjusted models, the magnitude of the association was slightly attenuated after accounting for socioeconomic factors, lifestyle factors, baseline diseases, medication use, and total energy intake, but the overall pattern remained stable (Table 2 and Figure 1).

**Table 2:** Association between sulfur microbial diet score and risk of ischemic stroke.

Exposure	Model1	P1	Model2	P2	Model3	P3
Q2 vs. Q1	1.01 (0.91, 1.11)	0.849	1.01 (0.92, 1.12)	0.827	1.02 (0.92, 1.13)	0.712
Q3 vs. Q1	1.08 (0.98, 1.20)	0.107	1.06 (0.96, 1.17)	0.242	1.07 (0.97, 1.19)	0.161
Q4 vs. Q1	1.21 (1.10, 1.34)	<0.001	1.11 (1.00, 1.22)	0.041	1.13 (1.02, 1.25)	0.019
Per 1-SD increase					1.04 (1.01, 1.08)	0.018
*P* for trend (Model 3)						0.013

Model 1 was adjusted for age, sex, and ethnicity.

Model 2 was further adjusted for BMI, Townsend deprivation index, education, income, employment, smoking status, alcohol intake, physical activity, diabetes, coronary heart disease, and hypertension.

Model 3 was further adjusted for total energy intake.

*P* for trend was calculated by assigning the median SMD value within each quartile and modeling it as a continuous variable.

SMD, sulfur microbial diet score; HR, hazard ratio; CI, confidence interval; BMI, body mass index; CHD, coronary heart disease.

**Figure 1:**
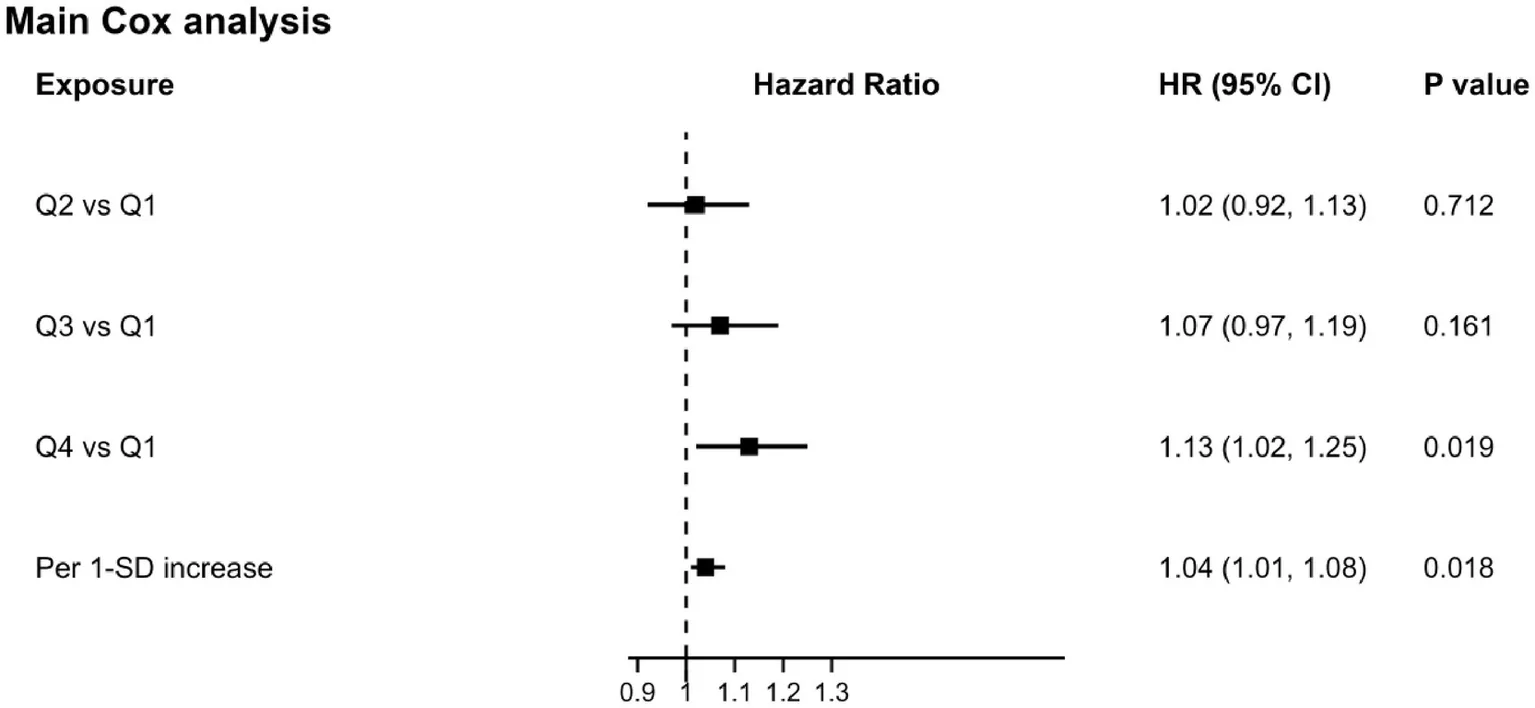
Association between the sulfur microbial diet score and risk of ischemic stroke.

### 3.3 Dose–response relationship

Restricted cubic spline analysis was compatible with an approximately linear association between the SMD score and ischemic stroke, with no statistical evidence of nonlinearity (*P* for nonlinearity = 0.232; Figure 2).

**Figure 2:**
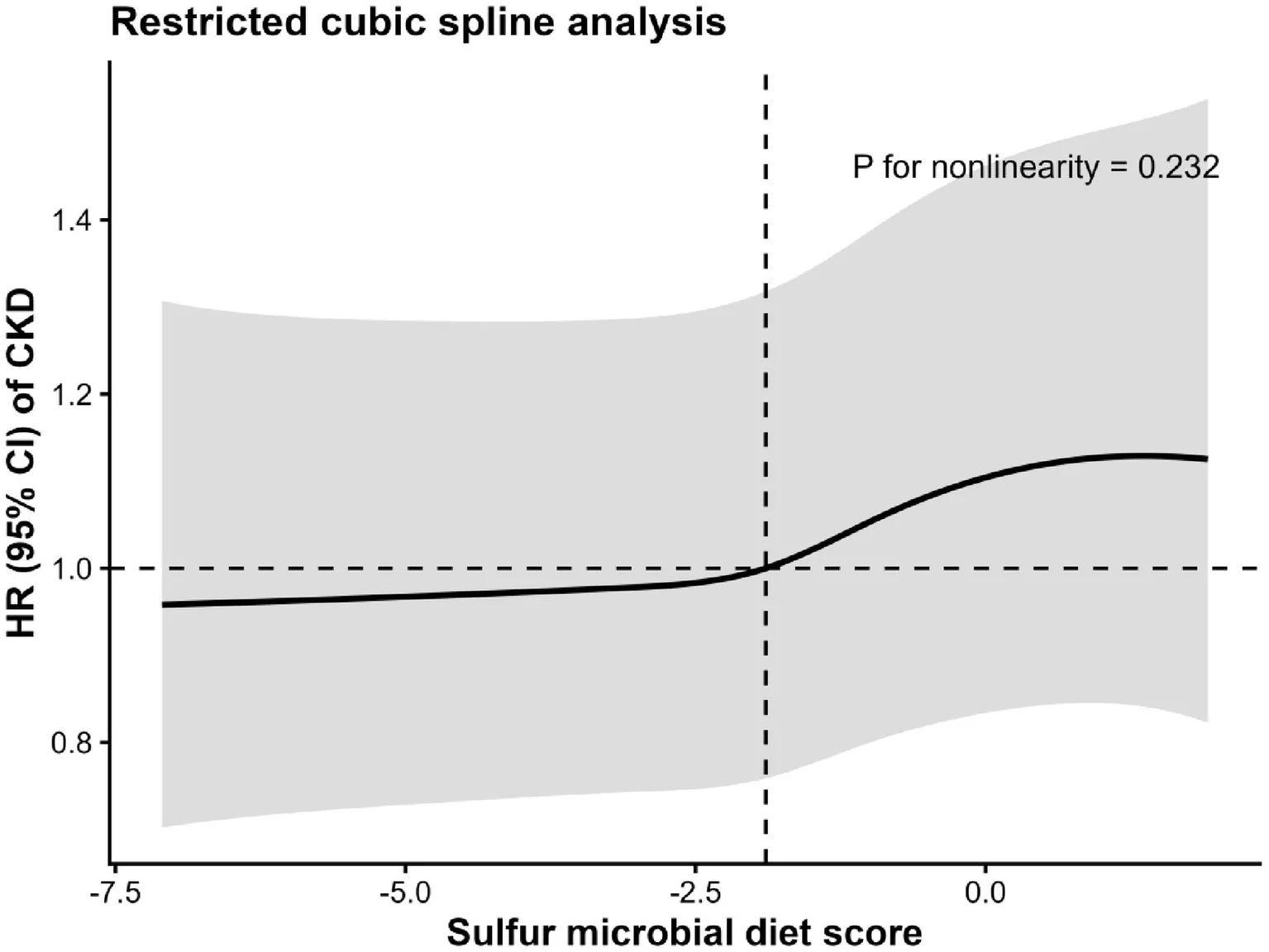
Restricted cubic spline association between the sulfur microbial diet score and risk of ischemic stroke.

### 3.4 Subgroup analyses

Subgroup analyses showed that the direction of the association between the SMD and ischemic stroke was generally consistent across strata of age, sex, smoking, and alcohol drinking, and most interaction tests were not statistically significant. Among participants aged ≥60 years, the HR for Q4 versus Q1 was 1.13 (95% CI: 1.00, 1.28; *p* = 0.043). Among women, the association was more pronounced, with an HR of 1.19 (95% CI: 1.01, 1.40; *p* = 0.038). Notably, stratified analysis by ethnicity showed that the SMD was significantly positively associated with ischemic stroke risk among White participants (HR = 1.16, 95% CI: 1.05, 1.29; *p* = 0.003), whereas an inverse association was observed among non-White participants (HR = 0.48, 95% CI: 0.28, 0.83; *p* = 0.009), with a significant interaction by ethnicity (*P* for interaction = 0.003). These findings suggest that the association between the SMD and stroke risk may be modified by ethnicity (Supplementary Figure S2 and Supplementary Table S2).

### 3.5 Sensitivity analyses

Sensitivity analyses yielded generally consistent point estimates. After excluding ischemic stroke events within the first 2 years, the HR was 1.12 (95% CI: 1.01, 1.24; *p* = 0.031). Among participants with at least two valid 24-h recalls, the estimate remained above 1 but was not statistically significant (HR = 1.08, 95% CI: 0.93, 1.24; *p* = 0.310). After excluding participants with baseline diabetes or coronary heart disease, the HR was 1.12 (95% CI: 1.00, 1.25; *p* = 0.045) (Supplementary Figure S3 and Supplementary Table S3).

### 3.6 Substitution analysis

In substitution models, replacing low-calorie beverages with an equivalent energy intake of other vegetables yielded an HR of 0.90 (95% CI: 0.81, 1.01; *p* = 0.063). Replacing sweets/desserts with other vegetables was not statistically significant (HR = 0.99; *p* = 0.371), and replacing sweets/desserts with low-calorie beverages also did not reach statistical significance (HR = 1.10, 95% CI: 1.00, 1.21; *p* = 0.078) (Table 3).

**Table 3:** Substitution analysis of sulfur microbial diet components and risk of ischemic stroke.

Substitution	HR 95% CI	*p*-value
With other vegetables (replace low-calorie drinks)	0.90 (0.81, 1.01)	0.063
With other vegetables (replace sweets and desserts)	0.99 (0.97, 1.01)	0.371
With low-calorie drinks (replace sweets and desserts)	1.10 (1.00, 1.21)	0.078

Hazard ratios (HRs) and 95% confidence intervals (CIs) were estimated using Cox proportional hazards models fitted separately in five multiply imputed datasets; regression coefficients and corresponding variance estimates were pooled using Rubin’s rules. Substitution models estimated the effect of replacing one dietary component with another while holding total intake constant.

## 4 Discussion

In this large prospective cohort, a higher SMD score was associated with a higher hazard of incident ischemic stroke over a mean follow-up of 11.45 years, and the dose–response pattern was approximately linear. Sensitivity analyses yielded directionally consistent estimates. Exploratory subgroup analyses suggested that the association varied by ethnicity. Substitution estimates were not statistically significant at the conventional 0.05 threshold.

Although the primary association was statistically significant in the fully adjusted model, the effect estimate was modest (HR per 1-SD = 1.04) and showed some sensitivity to exposure measurement precision. The estimate remained above 1 but lost statistical significance after restricting to participants with at least two dietary recalls (HR = 1.08; 95% CI 0.93–1.24; *p* = 0.310). These findings do not invalidate the primary result — the direction of association was consistently positive across all sensitivity analyses — but they indicate that the association is modest and may be influenced by exposure measurement precision. The overall pattern across sensitivity analyses supports the directional association, while the reduced precision in some subsets highlights the need for replication in larger cohorts with more repeated dietary measurements.

The major findings of this study are broadly consistent with previous research on gut microbiota-related dietary patterns and adverse cardiometabolic outcomes. Prior studies have linked sulfur microbial dietary patterns with colorectal cancer, all-cause mortality, and altered circulating metabolic profiles (8, 16), suggesting that such patterns may represent an unhealthy dietary structure characterized by inflammatory and metabolic features. Mechanistically, the dietary components and metabolites implicated in the SMD may influence inflammation, metabolism, and vascular function (10, 11, 17, 18). First, the SMD is characterized by high intakes of processed meats and low-calorie beverages, together with insufficient intake of protective plant-based foods. This combination may create a gut ecological environment favorable to the expansion of sulfur-metabolizing microbes (10). Enhanced sulfur metabolism may increase exposure to sulfur-containing metabolites such as hydrogen sulfide, which in turn may impair the intestinal epithelial barrier, increase intestinal permeability, and trigger chronic low-grade inflammation. In addition, the generally poor dietary quality reflected by the SMD may promote obesity, insulin resistance, and dyslipidemia, all of which are major underlying determinants of ischemic stroke (11). Moreover, the SMD may also contribute to stroke risk by enhancing oxidative stress, impairing vascular endothelial function, and aggravating lipid peroxidation (16–18), thereby facilitating plaque formation, destabilization, and thrombosis along the pathophysiological continuum from dietary exposure to stroke onset.

The findings are broadly consistent with research linking sulfur microbial dietary patterns with adverse metabolic profiles. The SMD is characterized by greater intakes of processed meat, low-calorie beverages, and liquor and lower intakes of several plant foods. Such a pattern may be associated with obesity, insulin resistance, dyslipidemia, inflammation, oxidative stress, and endothelial dysfunction, which are established correlates of ischemic stroke. Whether these factors account for the observed association was not evaluated in the present study.

The food substitution analysis was exploratory and did not yield statistically significant results for any of the component substitutions examined. Replacing low-calorie beverages with an equivalent energy intake of other vegetables yielded an HR below 1 (HR = 0.90; 95% CI: 0.81, 1.01; *p* = 0.063), whereas substituting sweets/desserts with other vegetables (HR = 0.99; 95% CI: 0.97, 1.01; *p* = 0.371) or with low-calorie beverages (HR = 1.10; 95% CI: 1.00, 1.21; *p* = 0.078) also did not reach statistical significance. These exploratory findings do not constitute evidence for specific dietary substitutions and should not be interpreted as established preventive recommendations. The observed direction of effects, although not statistically significant, is broadly consistent with the central direction of current global dietary guidelines (19). At present, the World Health Organization, the European Society of Cardiology, the American Heart Association, and dietary guidelines in several countries all emphasize limiting processed meat and sugar-sweetened or artificially sweetened beverages while increasing the intake of vegetables, legumes, and whole grains (20, 21). However, existing guidelines mainly focus on broad dietary structure and have not yet incorporated gut microbiota-related specific dietary patterns into formal recommendations (20, 22).

Exploratory subgroup analyses suggested heterogeneity by ethnicity: the SMD was positively associated with ischemic stroke among White participants (HR = 1.16, 95% CI: 1.05, 1.29) but inversely associated among non-White participants (HR = 0.48, 95% CI: 0.28, 0.83). The non-White stratum was comparatively small, and the interaction arose from multiple exploratory comparisons; it therefore requires replication before informing population-specific recommendations. Associations appeared stronger among women and adults aged at least 60 years, but the corresponding interaction tests were not statistically significant. By contrast, the direction of association was generally consistent across smoking and alcohol drinking subgroups, with no significant interactions observed (23). However, the absence of significant interactions does not demonstrate independence of the SMD association from these factors; it only indicates that, within the power constraints of this study, the association did not differ significantly across these strata. The subgroup analyses, particularly the significant interaction by ethnicity, were exploratory in nature (post-hoc analyses). Therefore, although biologically intriguing, this finding requires confirmation in future studies specifically designed to test ethnic differences in the SMD-stroke association.

Mechanistic inferences from the SMD–stroke association remain speculative because gut microbiome composition and sulfur-containing metabolites were not measured. The SMD is a dietary index linked to sulfur-metabolizing gut microbial function.

This study has several strengths, including its large sample size, long follow-up duration, standardized outcome ascertainment, and the use of repeated 24-h dietary recalls to improve the stability of dietary exposure assessment. In addition, we examined not only the overall association but also the dose–response relationship, subgroup heterogeneity, and food substitution effects, providing a more comprehensive interpretation of the findings. Nevertheless, several limitations should be acknowledged. First, as an observational prospective study, residual confounding cannot be completely ruled out despite extensive adjustment and multiple sensitivity analyses; therefore, causal inference cannot be established directly. Second, although the SMD score has a clear biological rationale derived from a previously validated algorithm linking dietary components to sulfur-metabolizing gut microbiota, we did not directly measure gut microbiome composition or sulfur metabolites in our participants. The SMD score is a dietary index linked to sulfur-metabolizing gut microbial function rather than a direct biological measurement. As a result, our mechanistic interpretations—particularly regarding the role of gut microbial sulfur metabolism in stroke pathogenesis—remain inferential and hypothesis-generating, warranting future confirmation through studies incorporating shotgun metagenomic sequencing and targeted metabolomics. Third, participants are predominantly middle-aged and older White individuals, caution is warranted when generalizing these findings to other populations. We observed a significant ethnicity interaction—the association was stronger in White participants but attenuated in non-White participants—though this is exploratory given the small non-White sample size and requires validation in diverse cohorts. Fourth, metabolic and inflammatory factors that may lie between the SMD and ischemic stroke, such as adiposity, glycemia, and systemic inflammation, were not formally evaluated; studies with appropriate mediation designs and repeated biomarker measurements are needed to examine these potential pathways.

Overall, this prospective analysis found that greater adherence to the SMD was associated with a higher hazard of ischemic stroke, although the association was modest and showed some sensitivity to exposure measurement precision. The observed associations support further investigation of microbiota-related dietary patterns in cerebrovascular health, and replication in independent cohorts is warranted.

## Data Availability

The original contributions presented in the study are included in the article/Supplementary material, further inquiries can be directed to the corresponding author/s.
