# Supplementary material for: Sulfur microbial diet and ischemic stroke risk
Source: Front Nutr. 2026 Oct 22;13:1885612. doi: 10.3389/fnut.2026.1885612 (PMC13735799; doi:10.3389/fnut.2026.1885612)
Supplement: Supplementary file 1 [file Supplementary_file_1.DOCX]

**Supplementary Material**

**Supplementary Tables**

**Supplementary Table S1. Sensitivity analyses of the sulfur microbial diet score and incident ischemic stroke**

| **Analysis** | **HR (95% CI)** | **P value** |
| --- | --- | --- |
| Exclude events within first 2 years | 1.12 (1.01, 1.24) | 0.031 |
| Exclude participants with only one 24-h recall | 1.08 (0.93, 1.24) | 0.31 |
| Exclude baseline diabetes and CHD | 1.12 (1.00, 1.25) | 0.045 |

**Supplementary Table S2. Subgroup analyses of the sulfur microbial diet score and incident ischemic stroke**

| **Subgroup** | **Level** | **HR (95% CI)** | **P value** | **P for interaction** |
| --- | --- | --- | --- | --- |
| Age | <60 | 1.01 (0.85, 1.21) | 0.900 | 0.910 |
| Age | ≥60 | 1.13 (1.00, 1.28) | 0.043 |  |
| Sex | Female | 1.19 (1.01, 1.40) | 0.038 | 0.829 |
| Sex | Male | 1.09 (0.96, 1.24) | 0.200 |  |
| Race | White | 1.16 (1.05, 1.29) | 0.003 | 0.003 |
| Race | Non-white | 0.48 (0.28, 0.83) | 0.009 |  |
| Alcohol intake | Never | 1.20 (0.84, 1.73) | 0.318 | 0.826 |
| Alcohol intake | <3 times/week | 1.20 (1.03, 1.40) | 0.017 |  |
| Alcohol intake | ≥3 times/week | 1.06 (0.92, 1.22) | 0.444 |  |
| Smoking status | Never | 1.25 (1.08, 1.45) | 0.003 | 0.298 |
| Smoking status | Current/previous | 1.06 (0.92, 1.21) | 0.421 |  |

Supplementary Table S3. Operational definitions of hypertension and diabetes.

| **Definition component** | **Participants meeting criterion** | **Percent** |
| --- | --- | --- |
| Blood pressure medication | 37,873 | 18.1 |
| Measured BP >=140/90 mmHg | 96,403 | 46.1 |
| Hypertension composite | 109,284 | 52.3 |
| Doctor-diagnosed diabetes | 8,725 | 4.2 |
| Insulin use | 1,743 | 0.8 |
| HbA1c >=48 mmol/mol | 5,680 | 2.7 |
| Glucose >=7 mmol/L | 5,595 | 2.7 |
| Diabetes composite | 11,926 | 5.7 |

Note: Hypertension was defined as measured systolic blood pressure >=140 mmHg, measured diastolic blood pressure >=90 mmHg, or use of blood-pressure-lowering medication. Diabetes was defined as doctor-diagnosed diabetes, insulin use, HbA1c >=48 mmol/mol, or glucose >=7 mmol/L. Medication indicators contributed to these definitions and were not entered again as separate covariates in the same model.

**Supplementary Figures**

**Figure S1. Participant included flow diagram.**

**Figure S2. Subgroup analyses of the association between the sulfur microbial diet score and risk of ischemic stroke.**

**Figure S3. Sensitivity analyses of the association between the sulfur microbial diet score and risk of ischemic stroke.**
